# Malignant Phyllodes Tumor in Adolescent Females: A Report of Three Cases and Review of the Literature

**DOI:** 10.1155/cris/5578363

**Published:** 2026-05-25

**Authors:** James Yahaya J., Gift Bunyata S., Emmanuel Morgan D., Tonny Okecha, Emmanuel Othieno

**Affiliations:** ^1^ Department of Pathology, School of Health Sciences, Soroti University, Soroti, Uganda; ^2^ Department of Pathology, Uganda Cancer Institute (UCI), Kampala, Uganda, uci.or.ug

**Keywords:** adolescence, clinical presentation, malignant phyllodes tumor, prognosis

## Abstract

Malignant phyllodes tumors (MPTs) are quite a rare type of mesenchymal breast cancer that can mimic benign lesions such as fibroadenomas histologically and radiologically. They account for less than 1% of all breast cancers, and they usually occur commonly in younger and middle‐aged women. We report three cases of MPT with ages ranging between 15 and 20 years from the Eastern region of Uganda that were obtained consecutively at our institution for the period of 3 years (from 2023 to 2025). Case 1 was a 15‐year‐old female with 2 months history of a left breast mass that was painful, rapidly growing, and associated with ulceration; it was confirmed histologically to be MPT and was treated successfully with ifosfamide 390 mg, doxorubicin 48 mg, and dexamethasone 8 mg. Case 2 was a 19‐year‐old female with 6‐month history of a right breast mass that was associated with pain and tenderness, and the mass was confirmed histologically to be MPT though she died before the initiation of treatment. Case 3 was a 20‐year‐old female with 3‐month history of a left breast mass that was associated with intermittent pains. She also had a positive family history of breast cancer. Her mass in the breast was confirmed histologically to be MPT and she was also treated with ifosfamide 390 mg, doxorubicin 48 mg, and dexamethasone 8 mg. All three cases tested positive for vimentin, but case 2 also showed focally positive SMA, but all of them were triple negative with Ki67 expression ranging from 20% to 35%. MPTs are rare and are more likely to recur, and they tend to have rapid growth and metastasis. Timely and proper management of patients with MPT helps to prevent possibilities of metastasis and improve the prognosis of the patients.

## 1. Introduction

Phyllodes tumors (PTs), previously known as cystosarcoma phyllodes, are an extremely rare form of fibroepithelial tumors of the breast, which account for up to 0.9% of all fibroepithelial tumors and less than 1% of all breast tumors [1]. PTs rarely occur in adolescents, and the mean age at diagnosis is between 40 and 50 years, and the prevalence of malignant PTs (MPTs) ranges from 10% to 15% of all cases of PTs [2], and they tend to occur between 2 and 5 years later than benign ones [3]. Histologically, considering the aggressiveness of PTs, they are divided into three tumor grades: benign (60%–75%), borderline (13%–26%), and malignant (10%–20%) [4]. Clinically, the average tumor size is about 4 cm, with less than 10% of the tumors being larger than 10 cm. However, in some case reports, it was reported that the giant forms of PTs may have a tumor size that can range from 30 to 48 cm [5, 6].

This report describes three patients with MPT from a resource‐limited setting in sub‐Saharan Africa (SSA). This series highlights the adolescent biology and challenges associated with early detection and management in a resource‐limited setting as well as the rarity of the disease. Our study explains the fact that younger females in resource‐limited settings may develop rare breast cancers, including MPTs, and this may be associated with a significant delay of diagnosis. Furthermore, this report provides insights regarding emphasis on the fact that MPTs frequently develop among adolescents.

## 2. Case Reports

### 2.1. Case 1

**Figure 1:**
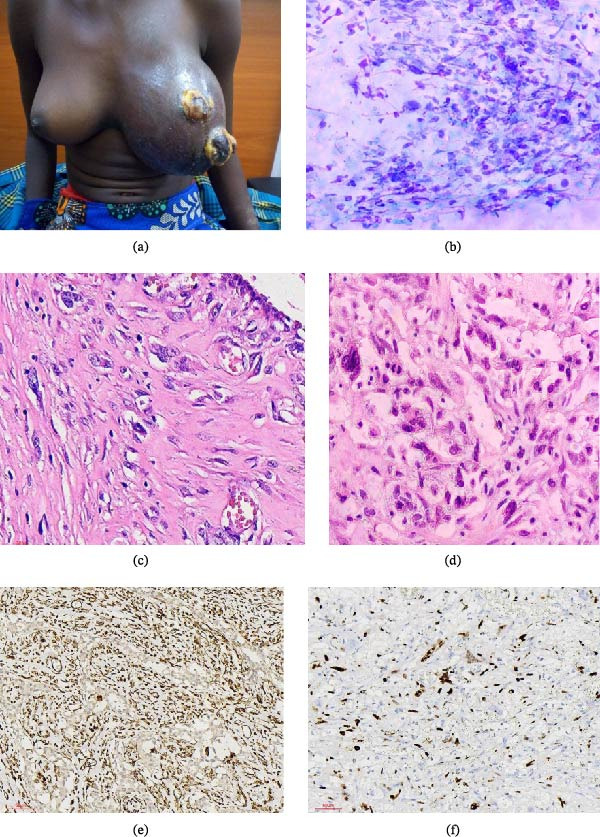
(a) Clinical picture of the patient showing swollen left breast with nodular ulcerating masses and (b) fine needle aspiration cytology (FNAC) microphotography. The smear shows proliferating hyperchromatic roundish and spindle‐shaped tumor cells (D‐Q stain, ×200). (c) Photomicrography showing proliferation of atypical spindle cells (H&E stains, ×400), (d) photomicrography showing tumor spindle‐shaped cells and some brisk mitosis (arrow) (H&E stains, ×400), (e) photomicrography image showing strongly positive intranuclear staining for vimentin antibody (IHC staining, ×40), and (f) photomicrography image showing positivity expression of Ki67 antibody (IHC staining, ×40).

We present the case of a 15‐year‐old female who was brought by her parents to the surgical outpatient clinic with a main complaint of a rapidly growing left breast mass for 2 months. The mass was initially painless; however, she experienced moderate pain over the past 6 days prior to seeking medical service. She reported no fever, nipple discharge, or weight loss. Her past medical history was uneventful, she neither reported a positive family history of breast or ovarian cancer nor a history of genetic disease. On physical examination, the patient looked generally unwell but stable. Her left breast was diffusely enlarged with two ulcerated and nodular foci (Figure 1a). However, no ipsilateral axillary, supraclavicular, or cervical lymphadenopathy was seen, and the contralateral breast was unremarkable. The patient was sent for fine needle aspiration cytology (FNAC), which was done and showed moderately cellular smears composed of sheets and strands of spindle cells with mild atypia, which were suspicious for a fibroepithelial tumor (Figure 1b).

Three days later, after obtaining results from FNAC, an incisional biopsy was done to confirm the diagnosis, which showed a diffuse tumor composed of proliferating sheets of malignant spindle‐shaped cells with moderate to marked atypia, and about 20 mitoses per 10 high‐power fields (HPFs) were seen (Figure 1c,d). The tumor was histologically confirmed to be MPT. A panel of immunohistochemistry (IHC) antibodies, including rabbit monoclonal antibodies of ER (clone SPI), PR (clone 1E2), HER‐2/neu (clone 4B5), and Ki67 (clone 30‐9) was done using the BenchMark IHC automated system and scored as per the American Society of Clinical Oncology–College of American Pathologists (ASCO/CAP) and the International Ki67 in Breast Cancer Working Group guidelines [7–9]. To confirm the diagnosis and rule out other differentials, an additional panel of IHC monoclonal primary antibodies testing, which consisted of antivimentin (clone V9), anti‐CD31 (clone JC70), anti‐CD34 (clone QBEnd/10), anti‐P63 (clone A4A), and anti‐S100 (clone 4C4.9), was done. Quality control for IHC antibody testing was ensured by following the recommended ASCO/CAP guidelines [10]. All specimens had a cold ischemia time of 30 and 45 min, and the specimens were serially sliced at 3 μm thickness and placed in neutral buffered 10% formalin to fix for a maximum of 72 h. All sections processed include a tumor with small normal breast tissues to serve as an internal positive control for the hormone receptors and HER2. All IHC slides, whether positive or negative, were reviewed by two independent pathologists. ER and PR were considered negative when there is no tumor cell nuclear staining or there was nuclear staining in <1% of tumor cells as per the guidelines. Internal positive controls, wherever present, stained positive for both ER and PR, confirming the true negative results. HER2 was considered negative score (0) when no membrane staining was seen in any tumor cells, or when weak/faint and incomplete membrane staining was seen in ≤10% of tumor cells. The Ki67 was scored visually as percentage according to the International Ki67 in Breast Cancer Working Group. Furthermore, a panel of antibodies was done, which showed strongly positive vimentin and 30% expression of Ki67 (Figure 1e,f). However, S100, ER, PR, HER2, BCL2, P63, CD31, and CD34 were all negative. All the antibodies were purchased from Ventana Medical Systems, Inc. (Arizona, USA).

The patient was referred to a national cancer center. The investigations done at the cancer center included a renal function test, liver function test and complete blood count, all of which were unremarkable. Additionally, a staging chest CT scan was also done, and it revealed a large heterogeneous dense lobulated solid mass with irregular margins, and the tumor measured 15.9 cm × 9.6 cm × 12.7 cm with necrotic changes. There were numerous chest wall and axillary lymph nodes of variable size (level I, II, and III) and the largest measured 2.8 cm in diameter. Ipsilateral supraclavicular lymph nodes were involved up to level V. However, there was no evidence of involvement to the lungs, and the heart and other great vessels appeared normal. The upper abdomen (liver, spleen, and pancreas) was unremarkable.

The patient was reassessed for excision of the tumor, and a modified radical mastectomy with surgical margins of >1 cm width was done together with axillary lymph node dissection (ALND) because the axillary lymph nodes were palpable and suspicious for malignancy on imaging. The mastectomy specimen received measured 23 cm × 19 cm × 11 cm with two ulcerated tumor nodules protruding on the surface, and the cut surface of the specimen showed a huge white‐tan and firm mass which measured 15.9 cm in its greatest diameter, and it was partially encapsulated. Histologically, the tumor cells were morphologically similar to the tumor cells examined from the incisional biopsy, and also the tumor margins of the mastectomy specimen were free from the tumor cells. There was no lymphovascular or perineural invasion, and all the five axillary lymph nodes showed reactive changes, and therefore a diagnosis of MPT was confirmed. Additionally, the surgical margins were free from tumor cells. The histologic criteria used to define malignancy included stromal overgrowth, mitoses, necrosis, nuclear atypia, and infiltrative tumor margins. The American Joint Committee on Cancer (AJCC) TNM classification for soft tissue sarcomas (8th edition) of 2017 was used in assigning the tumor stage of the patient [11] and as it was used in the report of Hauser et al. [12]. The tumor was classified as pT4 (T >15 cm), G3 (high grade), and pN0 (0/5‐no positive lymph node) (pT4G3pN0). After a follow‐up duration of 18 months which included a chest CT scan and clinical evaluation for possible recurrence or metastasis, the patient was found free of the tumor or significant complications of the chemotherapy drugs and currently she is clinically stable. During follow‐up surveillance for local recurrence or metastasis was done using a CT scan after every 6‐month, as it is recommended for the first 2 years.

### 2.2. Case 2

**Figure 2:**
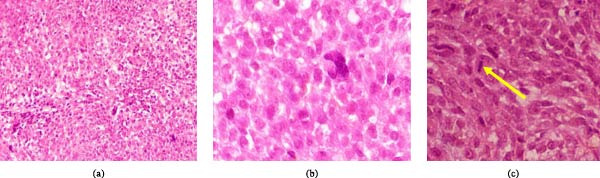
(a) Photomicrography showing proliferation of the two population of spindle and roundish tumor cells (H&E stains, ×100), (b) photomicrography showing an area with predominating roundish tumor cells with course nuclear chromatin (H&E stains, ×400), and (c) photomicrography showing predominating spindle cells and mitosis (arrow) (H&E stains, ×400).

The patient was a 19‐year‐old female with a 6‐month history of right breast swelling, which was associated with pain and tenderness. Her past medical history was uneventful, and she had neither family history of any cancer nor genetic disease. On physical examination, she was fairly looking with breast asymmetry due to a swelling in the right breast. On palpation, the swelling was hard, nodular, and fixed. The overlying skin of the breast and the nipple was normal. An incisional biopsy was taken from the mass, which was friable and easily bleeding on touch. Microscopically, the breast tissue sections showed marked proliferating mesenchymal tumor cells. The tumor cells were hyperchromatic, spindle and roundish with fine to coarse nuclear chromatin, and they had a tendency to form fibrovascular cores. Nuclear pleomorphism was marked, and some other tumor cells had prominent nucleoli. Brisk mitoses of about 30 per 10 HPFs were seen together with giant tumor cells (Figure 2a–c). IHC tests showed strongly positive vimentin, which is suggestive of mesenchymal origin for the tumor, focally positive SMA, and Ki67 expression of 35%. However, the case was triple negative, and therefore a diagnosis of MPT was confirmed based on positivity for vimentin and SMA antibodies. Unfortunately, the patient died 2 months later after the histological report from the incisional biopsy was produced before she had started management due to challenges with money for fare from her remote home place and lack of awareness from the parents of knowing that she could be helped to receive treatment at the cancer institute.

### 2.3. Case 3

**Figure 3:**
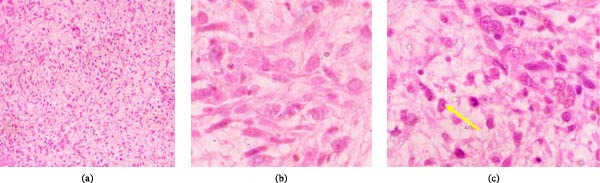
(a) Photomicrography showing proliferation of hyperchromatic spindle tumor cells (H&E stains, x100), (b) photomicrography showing tumor spindle and roundish pleomorphic tumors cells and some with prominent nucleoli (H&E stains, x400), and (c) the tumorous area with many mitoses (arrow) (H&E stains, x400).

A 20‐year‐old female with a 3‐month history of a left breast mass associated with intermittent pains. Her past medical history was uneventful, though she had a positive family history of breast cancer (her aunt died of breast cancer), but she had no history of genetic disease. On local examination, there was a swelling in the left breast, which was hard, roundish, and fixed. The overlying skin of the breast and the nipple were normal, and there was no nipple discharge. Also, there were no palpable axillary lymph nodes. An incisional biopsy was taken and submitted for histological evaluation. Microscopically, the specimen showed a solid diffuse tumor composed of proliferating spindle‐shaped malignant tumor cells with marked nuclear atypia. Some roundish to ovoid tumor cells with prominent nucleoli were also seen. A mitotic count of about 15 per 10 HPFs, multinucleated giant tumor cells, and necrosis were also seen (Figure 3a–c).

After histological confirmation of the MPT diagnosis, the patient was referred to the national cancer centre, where laboratory investigations, imaging, and management of the patient were carried out. A complete blood count mainly revealed mild anemia (Hb level 10 gm/dL) and thrombocytopenia (platelet count 135 × 10^3^/µL). The liver and renal function tests were normal, but erythrocyte sedimentation rate (ESR) >30 mm/hr and C‐reactive protein (CRP) (14 mg/L) were both raised. A chest X‐ray was done, which did not show abnormality. Also, a chest CT scan was done, which was normal. Radical mastectomy together with surgical margins of >1 cm and ALND were done. Cut surface of the submitted mastectomy specimen showed a white‐tan and firm mass which measured 8.4 cm in its greatest diameter and was partially encapsulated. The axillary tail measured 4.7 cm in length, from which four lymph nodes were dissected, with the largest measuring 3 cm in diameter.

Microscopically, the histological features from the tumor resembled those that were seen in the incisional biopsy, which was done initially, and all the three axillary lymph nodes were reactive. The assigned AJCC TNM stage for the patient was pT2, G3, pN0 (0/4). The surgical margins were free from tumor cells. Additionally, IHC tests showed positive vimentin and Ki67 expression of 20%. However, this case was also triple negative, and therefore a diagnosis of MPT was confirmed. After a duration of 2 weeks postsurgery, she was started on three cycles of adjuvant chemotherapy, which consisted of ifosfamide 410 mg, doxorubicin 54 mg, and dexamethasone 12 mg for 3 months based on having a large tumor size, high tumor grade, and stromal overgrowth. She completed successfully her chemotherapy regimen, though with episodes of some drug complications such as nausea, vomiting, and fatigue. She was followed up after a period of 11 months, and her condition is currently stable without local recurrence or metastasis. The surveillance method during the follow‐up period of the patient was done as in case 1. Table 1 summarizes the age of the patient, tumor size, status of surgical margins, nodal status, mitotic index, adjuvant therapy, follow‐up duration, and current status.

**Table 1:** Summary of the clinicopathologic features and outcome of the patients.

Cases	Age (years)	Tumor size (cm)	Surgical margins	Nodal status	Mitotic index per 10 HPFs	Adjuvant therapy	Follow‐up duration (months)	Current status
1	15	15.9	Negative	Reactive	20	Chemotherapy	18	Alive
2	19	–	–	–	30	–	–	Dead
3	20	8.4	Negative	Reactive	15	Chemotherapy	11	Alive

## 3. Discussion

Clinically, MPT usually presents as a rapidly growing unilateral painless breast mass, and the skin overlying the tumor may have a blue discoloration with dilated veins, but ulceration is very rare [13]. The vast majority of patients with MPT have no known history of predisposing factors; however, some patients with a history of fibroadenomas have been reported [14–17]. Considering the presence of ulceration and possibility of having a history of fibroadenoma, the case described in this report did not have both, which indicates that the pathogenesis of PTs may be diverse.

MPT is microscopically characterized by the combination of marked nuclear atypia of stromal cells, overgrowth of the stromal component due to absence of epithelial component in one low‐power microscopic field, increased mitotic activity (>10 per 10 HPF), and infiltrative borders [4]. For the patients described in the present study, the mitotic count was ranging between 15 and 30 per HPFs. Additionally, a diagnosis of MPT can also be made when malignant heterologous elements are present, including liposarcoma, angiosarcoma, osteosarcoma, chondrosarcoma, and rhabdomyosarcoma [18] even in the absence of other features [19]. MPTs are usually characterized by a high degree of nuclear atypia and stromal overgrowth with increased mitosis, stromal hypercellularity, and infiltrative tumor borders [20]. All three cases in our study have marked nuclear pleomorphism.

MPTs have a high tendency to recur, and almost 30% of MPTs have the possibility of recurring between 2 and 3 years following removal of the primary tumor [21]. For example, in the study of Mangi et al. [22], it was found that all five cases that had local recurrence, had a surgical margin of less than 1 cm (10 mm). For that reason, overcoming the possibility of recurrence, wide local excision (WLE) of the surgical margin of greater than 1 cm has been found so useful. Our two patients (case 1 and 3) among whom mastectomy was done, both had no recurrence at the end of 1 year postsurgery probably because a surgical margin of greater than 1 cm was achieved during surgery.

MPTs do not commonly metastasize, and one study reported that their rate of metastasis is ~25% [23] and they almost always spread by the hematogenous route [20, 21] with the highest chance of metastasizing to the lungs. Similarly, one of our cases (case 1) had lung metastasis, but she attained complete remission. Lymph node metastasis for MPTs is rare and ALND should be restricted only for rare patients showing palpable or suspicious lymph nodes on imaging [24]. Studies have shown that routine ALND among patients with MPTs is not recommended as part of management because MPTs spread hematogenously instead of lymphatic vessels [25, 26]. Most of palpable or radiologically suspicious axillary lymph nodes in patients with MPTs are normal or reactive, and metastasis to axillary lymph nodes has been found to be less than 1% [27]. And axillary lymph node metastasis in MPTs is associated with poor prognosis and short overall survival [23, 25]. Our patients in whom there was ALND (cases 1 and 3), none of them had lymph node metastasis. The role of either sentinel lymph node biopsy or ALND in the subpopulation of patients with MPTs is usually reserved for patients with tumor stages 1 and 2 [28].

Currently, there is no available specific treatment for MPTs due to a lack of large follow‐up studies caused by the rarity nature of the disease. The current standard treatment for MPTs is surgical excision with a minimum surgical margin of greater than 1 cm [29] for the reason of preventing local recurrence. In a systematic meta‐analysis study by Yu et al. [30] it was found that all patients with MPT that had a surgical margin not greater than 1 cm developed local recurrence. Prevention of local recurrence was not witnessed in our two cases (cases 1 and 3) partly because of surgical margins greater than 1 cm. Adjuvant chemotherapy and/or radiation therapy is not usually recommended for patients with MPTs due to a lack of evidence in improving the overall survival of the patients with such treatment options [31]. This was also observed in the two cases (cases 1 and 3) that did not develop recurrence or metastasis for a period of 1 year even without involving adjuvant chemotherapy and/or radiotherapy, especially for case 1. Although there is contradicting information regarding the benefits of neoadjuvant and adjuvant chemotherapy, the use of neoadjuvant chemotherapy for sarcomas has been emphasized in the past [32]. Indications for adjuvant chemotherapy for patients with MPTs include presence of metastasis, stromal overgrowth, difficulty in achieving wide surgical margins (>1 cm), and presence of local recurrence [23].

It has been found that radiotherapy is of benefit in controlling development of local recurrence. Furthermore, radiotherapy is useful when it is not possible to obtain wide surgical margins (>1 cm) [33], when breast‐conservative therapy (BCT) has been done instead of mastectomy, and for primary tumor with size greater than 5 cm [34], larger tumor sizes more than 10 cm [35], and high tumor grades [36]. It is important to ensure that wide excision of the primary tumor is done in patients with MPTs, even benign ones, to prevent the development of local recurrence. Our patients (cases 1 and 3) did not need radiotherapy because WLE of greater than 1 cm was achieved in both patients. Despite radiotherapy having the ability to control local recurrence in patients with MPTs, there is no evidence regarding its ability to improve the overall survival of the patients [37].

The age of the patients with MPTs has been found to contribute to variation in prognosis between young patients (children and adolescents) and adults. All three patients reported in this series were adolescents, and none of them had distant metastasis or lymph node involvement at the time of diagnosis. Also, of the two patents that were followed up, none of them had developed local recurrence or progression of the disease after approximately 1 year. The age distribution of the patients in this study was not different from the age of adolescent patients reported in other studies. For example, in the study of Gupta et al. [26] which included four patients with PTs, two of them had MPTs with age 17 and 20 years. This indicates that children and adolescents are more likely to develop MPTs compared to adults; however, the overall survival of children and adolescent patients with MPTs is superior to that of adults [38, 39]. There are no clear reasons which may help to explain the difference in prognosis of MPTs between adults and children or adolescents. Some factors such as less involvement of axillary lymph nodes in children and adolescents could be a contributing factor for better prognosis.

Financial constraints remain one of the major challenges affecting timely initiation of treatment among cancer patients in resource‐limited settings. This contributes to prolonged hospitalization and high mortality. For example, our patient (case 2) died even before the initiation of treatment because of financial issues that prevented her from starting treatment timely, leading to her death. Oncology service scarcity in most resource‐limited settings is still a major challenge, which significantly results in delays of diagnosis and treatment initiation [40]. Challenges to affordable cancer services in SSA have been reported as the major factor for the observed high mortality among cancer patients [41]. A number of socioeconomic barriers contribute to access inequities towards oncology services; such barriers include geographical distribution of services and transportation challenges, inadequate insurance and financial constraints, limited social support networks, and limited resources for social care needs, among others [42]. In SSA there is an extreme shortage of pathologists, and reports indicate that one pathologist in SSA attends 500,000 people compared to one pathologist per 15,000 or 20,000 people in high‐income countries [43]. This contributes to diagnostic delays as a result of prolonged turnaround time (TAT). Furthermore, a few pathologists available in SSA countries are usually confined to large cities, leaving rural areas without pathologists, which also contributes to diagnostic delays [44].

Other factors have also been found of prognostic value apart from lymph node involvement, metastasis, stromal cell atypia, presence of a heterologous component, and age of the patients. Such factors include a high mitotic count (≥10 mitoses per 10/HPFs), a positive surgical margin (<1 cm), and tumor size. In one study, it was reported that mitotic count is more prognostic among patients with MPTs compared to tumor size [22, 39]. It has also been observed that increased mitotic count is associated with stromal overgrowth and formation of infiltrative margins, all of which contribute to worsening the prognosis. The mitotic count has also been useful in distinguishing the benign from the malignant or borderline form of PTs, whereby benign and borderline PTs have <5 mitoses [45] and 5–9 [46] mitoses per 10 HPFs, respectively. Di Liso et al. [46] also reported that high mitotic count (≥10 mitoses/10 HPFs) was associated with malignancy of PTs. Another study which was done in India reported that having ≥10 mitoses/10 HPFs among patients with MPTs was significantly associated with reduced overall survival, unlike tumor size [47]. Also, all our patients in this study had a mitotic count >10/HPFs, indicating that they had an aggressive form of the disease. Surveillance of patients during the follow‐up period requires evaluation of local recurrence or metastasis after every 6 months for the first 2 years [48]. Ultrasound may be used during evaluation for patients with a history of BCT. However, for the reason of avoiding missing deep lesions, CT scan or MRI is advised due to high resolution. Then after the first 2 years of follow‐up, surveillance is advisable to be done annually for a period of 5 years or even 10 years [49].

## 4. Conclusion

This report of three cases of MPTs in female adolescents’ patients with one fatal outcome signifies the importance of including these tumors in differential diagnoses in adolescents’ and young adults’ females presenting with breast masses. Despite the rare nature of these tumors, this study highlights the crucial role of FNAC for preliminary diagnosis and early detection of these tumors, especially in a resource‐limited settings, and the role of wide surgical resection with >1 cm clear margins and close follow‐up in reducing the chances of local recurrence and improving overall survival in patients with this rare entity.

## Funding

No funding was received for the research, authorship, and/or publication of this article.

## Ethics Statement

Ethical approval was waived by the chairperson of the Ethical Research Committee. Preparation of this report was based on the ethical standards of both international and institutional research regulations.

## Consent

Written informed consent was obtained from the patients except for case 1 whose consent was obtained from her parents for publication of this case report and any accompanying images. Copies of the written consents are available for review by the Editor‐in‐Chief of this journal.

## Conflicts of Interest

The authors declare no conflicts of interest.

## Data Availability

The data that support the findings of this study are available upon request from the corresponding author. The data are not publicly available due to privacy or ethical restrictions.
